# Phyllosphere epiphytic and endophytic fungal community and network structures differ in a tropical mangrove ecosystem

**DOI:** 10.1186/s40168-019-0671-0

**Published:** 2019-04-09

**Authors:** Hui Yao, Xiang Sun, Chao He, Pulak Maitra, Xing-Chun Li, Liang-Dong Guo

**Affiliations:** 1 State Key Laboratory of Mycology, Institute of Microbiology, Chinese Academy of Sciences, Beijing, 100101 People’s Republic of China; 2 College of Life Sciences, University of Chinese Academy of Sciences, Beijing, 100049 People’s Republic of China; 3 Institute of Medicinal Plant Development, Chinese Academy of Medical Sciences and Peking Union Medical College, Beijing, 100193 People’s Republic of China

**Keywords:** Endophytic fungi, Epiphytic fungi, Illumina MiSeq sequencing, ITS2, Mangroves, Network structure

## Abstract

**Background:**

Revealing the relationship between plants and fungi is very important in understanding biodiversity maintenance, community stability, and ecosystem functioning. However, differences in the community and network structures of phyllosphere epiphytic and endophytic fungi are currently poorly documented. In this study, we examined epiphytic and endophytic fungal communities associated with the leaves of six mangrove species using Illumina MiSeq sequencing of internal transcribed spacer 2 (ITS2) sequences.

**Results:**

A total of 635 operational taxonomic units (OTUs) of endophytic and epiphytic fungi were obtained at a 97% sequence similarity level; they were dominated by *Dothideomycetes* and *Tremellomycetes*. Plant identity had a significant effect on the OTU richness of endophytic fungi, but not on epiphytic fungi. The community composition of epiphytic and endophytic fungi was significantly different, and plant identity had a greater effect on endophytic fungi than on epiphytic fungi. Network analysis showed that both epiphytic and endophytic network structures were characterized by significantly highly specialized and modular but lowly connected and anti-nested properties. Furthermore, the endophytic network had higher levels of specialization and modularity but lower connectance and stronger anti-nestedness than the epiphytic network.

**Conclusions:**

This study reveals that the phyllosphere epiphytic and endophytic fungal communities differ, and plant identity has a greater effect on the endophytic fungi than on epiphytic fungi. These findings demonstrate the role of host plant identity in driving phyllosphere epiphytic and endophytic community structure.

**Electronic supplementary material:**

The online version of this article (10.1186/s40168-019-0671-0) contains supplementary material, which is available to authorized users.

## Background

The interaction between plant and microbial communities drives the maintenance of biodiversity, community stability, and ecosystem functioning [1]. Phyllosphere fungi are an important component of microbial communities; they include both epiphytic fungi inhabiting leaf surfaces and endophytic fungi living asymptomatically within leaves, and are of high species diversity and play major roles in ecosystem functions [2–5]. For example, endophytic fungi can promote plant growth and resistance to biotic and abiotic stresses, such as pathogens, drought, and salinity [2, 6, 7]. In addition, epiphytic and endophytic fungi can contribute to leaf litter decomposition and play an important role in recycling carbon and nutrients in ecosystems [8–10]. Thus, elucidating the relationship between plants and phyllosphere epiphytic and endophytic fungi is of great importance if we are to understand biodiversity maintenance, community stability, and ecosystem functioning.

Phyllosphere epiphytic and endophytic fungi occupy two distinct microenvironments: epiphytic fungi are in contact with the external environment, and depend on nutrients deposited on leaves from the atmosphere or those exuded from leaves [11], whereas endophytic fungi are in contact with the plant’s inner environment and absorb nutrients from host tissues [12]. Plants are therefore presumably able to exert more control over the fungal colonization of internal tissues than that of exterior surfaces [13]. For example, some studies have shown that plant identity significantly affects endophytic fungal diversity and community composition [14–18]. By contrast, some studies have demonstrated that plant identity has no or weak effects, on epiphytic fungal diversity and community composition [19, 20]. Furthermore, previous studies have shown that the diversity and community composition of phyllosphere epiphytic and endophytic fungi are different [13, 21–23].

Disentangling the interactions of plants and fungi using ecological network analysis can give us a deeper understanding of the mechanisms underlying species coexistence and ecosystem stability [24, 25]. Among the main structural properties of networks, the nestedness concept describes a particular pattern of interaction in which the more specialist species interact only with proper subsets of those species interacting with the more generalists [26]. On the other hand, modularity is a measure of the extent to which the network is structured as cohesive subgroups of nodes (modules), in which the density of interactions is higher within subgroups than among subgroups [27]. A nested network architecture can make a community more robust to random extinctions [28] and enhance biodiversity by reducing interspecific competition and facilitating species coexistence [29]. By contrast, modular organization can increase overall network stability, particularly by limiting the impact of perturbations within a single module and minimizing impacts on other modules, and therefore buffering communities against secondary extinctions following disturbance [30].

Recently, based on re-analysis of previously published datasets of biotrophic plant-fungal associations, it has been suggested that nestedness decreases but modularity increases with increasing specificity [25], whereas nestedness increases but modularity decreases with increasing connectance [31]. However, most previous network studies on fungi have focused mainly on belowground mycorrhizal mutualistic networks, which vary among different mycorrhizal types and different ecosystems [32–40]. By contrast, the network structure of plants with endophytic and epiphytic fungi is less well documented [31, 41, 42]. For example, Ikeda et al. [41] revealed that the network of endophytic *Xylariaceae* and woody plants in Japan showed significant specialization. Chagnon et al. [42] found that the endophytic networks of plants were less nested, less connected, and more modular than endolichenic networks across five sites in North America and suggested that plant hosts could select more strongly than lichens for a specific subset of fungal partners. In addition to interaction types, interaction intimacy (i.e., the degree of biological associations between partners) can lead to differences in network organization; that is, increasing intimacy can promote specialization, leading to networks characterized by compartmentalization, whereas weak intimacy can lead to nested networks [43].

Mangrove forests are unique intertidal ecosystems confined to the subtropical and tropical regions and they contain about 70 plant species of 27 genera in 20 families, occupying ca. 137,760 km^2^ all over the world [44]. Mangrove forests have very important ecological and economical values, such as promoting sludge sedimentation, protecting coastlines from hurricanes, and providing breeding sites for many animal species and materials (e.g., fuel, timber, and tannins) for human [44]. Although many studies have been conducted on the endophytic fungi of mangroves [45, 46], there have been no studies comparing endophytic and epiphytic fungal communities.

For this reason, in order to reveal differences in the community and network structures of epiphytic and endophytic fungi, we examined epiphytic and endophytic fungal communities associated with the leaves of six mangrove species (*Aegiceras corniculatum*, *Avicennia marina*, *Bruguiera gymnorrhiza*, *Kandelia candel*, *Rhizophora stylosa*, and *Excoecaria agallocha*) in south China using Illumina MiSeq sequencing techniques. Because epiphytic and endophytic fungi occupy two distinct micro-habitats, resulting in the intimacy of interaction between plants and endophytic fungi being stronger than that between plants and epiphytic fungi, we therefore hypothesize that the communities of epiphytic and endophytic fungi differ, and plant identity has a greater effect on endophytic fungi than on epiphytic fungi. We also used network analysis to test the hypothesis that the endophytic network is more specialized and modular but less nested and connected than the epiphytic network in a mangrove system.

## Results

### Characterization of Illumina sequencing data

**Fig. 1:**
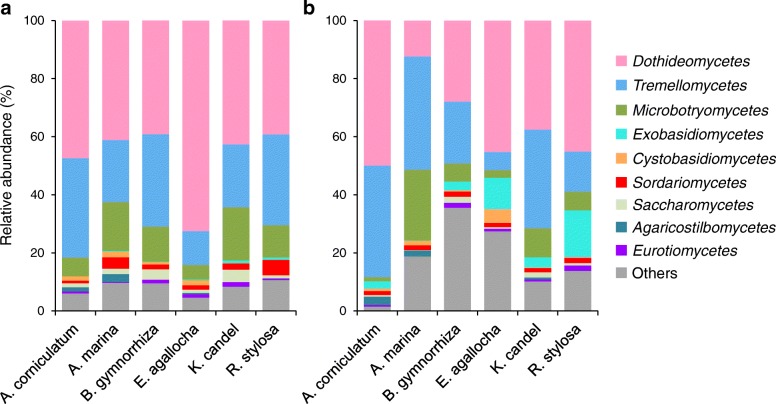
Relative abundance of fungi at the class level in mangrove species. **a** Epiphytic fungi. **b** Endophytic fungi. The fungal class represents < 0.5% of the total reads of epiphytic and endophytic fungi and fungi are not identified to class level were all assigned to “Others”. *A*. *corniculatum*, *Aegiceras corniculatum*; *A*. *marina*, *Avicennia marina*; *B*. *gymnorrhiza*, *Bruguiera gymnorrhiza*; *E*. *agallocha*, *Excoecaria agallocha*; *K*. *candel*, *Kandelia candel*; *R*. *stylosa*, *Rhizophora stylosa*

After removing 1,221,272 sequences that belong to low-quality, non-fungi, potential chimeras and singletons, the remaining non-chimeric fungal internal transcribed spacer 2 (ITS2) sequences (3,083,632 in total) were clustered into 1160 non-singleton operational taxonomic units (OTUs) at a 97% sequence similarity level. Of these 1160 OTUs, 826 (2,525,065 reads) were identified as fungal. From this dataset, we removed the OTUs with fewer than 10 reads, leaving 639 fungal OTUs. As the fungal read numbers ranged from 6827 to 49,955 across the 96 samples, the read number was normalized to 6827, resulting in a normalized dataset comprising 635 fungal OTUs (655,392 reads). The fungi represented included 456 *Ascomycota*, 169 *Basidiomycota*, 1 *Cryptomycota*, and 9 unknown fungi (Additional file 1: Table S1), and dominated by *Dothideomycetes* and *Tremellomycetes* both in the epiphytic and endophytic fungal communities, with varying relative abundance among different mangrove species (Fig. 1a, b). The 42 relatively abundant OTUs (> 1000 reads) accounted for 88.4% of the reads for epiphytic fungi, and the 41 relatively abundant OTUs (> 1000 reads) accounted for 87.3% of the reads for endophytic fungi (Additional file 2: Figure S1). For both epiphytic and endophytic fungi, rarefaction curves for the observed OTUs in the six plant species showed no signs of reaching asymptotes, suggesting that further sampling would recover more OTUs (Additional file 2: Figure S2).

### Richness of epiphytic and endophytic fungi

**Fig. 2:**
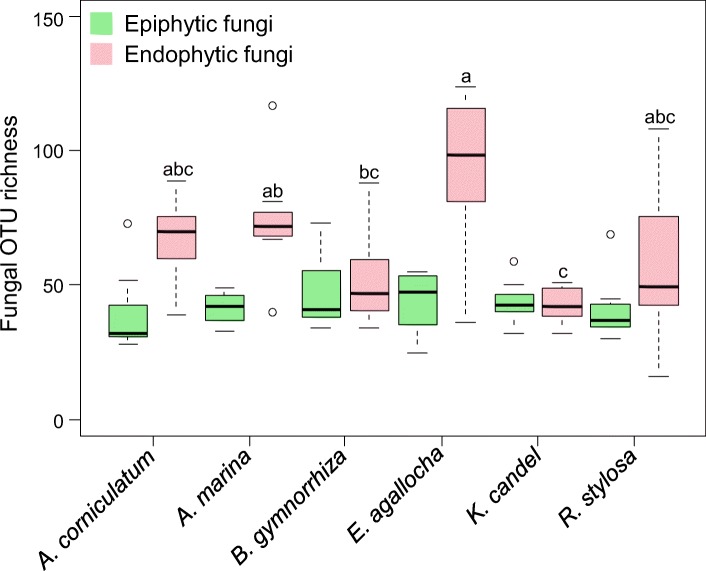
Operational taxonomic unit (OTU) richness of epiphytic and endophytic fungi in mangrove species. The black line inside each box represents the median value (*n* = 8). Kruskal–Wallis test and one-way ANOVA revealed that plant identity had a significant effect on the OTU richness of endophytic fungi (*χ*^2^ = 17.849, *P* = 0.003), but not on that of epiphytic fungi (*F*_5,42_ = 0.654, *P* = 0.660). Bars without shared letters indicate significant differences in the OTU richness of the endophytic fungi among mangrove species determined by Conoverʼs test at *P* < 0.05. *A*. *corniculatum*, *Aegiceras corniculatum*; *A*. *marina*, *Avicennia marina*; *B*. *gymnorrhiza*, *Bruguiera gymnorrhiza*; *E*. *agallocha*, *Excoecaria agallocha*; *K*. *candel*, *Kandelia candel*; *R*. *stylosa*, *Rhizophora stylosa*

The OTU richness of epiphytic and endophytic fungi respectively was 39.0 ± 15.2 and 67.4 ± 15.4 in *A*. *corniculatum*, 41.5 ± 5.7 and 74.0 ± 21.1 in *A*. *marina*, 47.0 ± 14.0 and 52.0 ± 17.3 in *B*. *gymnorrhiza*, 44.1 ± 11.0 and 93.9 ± 28.9 in *E*. *agallocha*, 43.6 ± 7.9 and 42.8 ± 6.6 in *K*. *candel*, and 41.0 ± 12.2 and 57.4 ± 29.8 (means ± SD) in *R*. *stylosa* (Fig. 2). Kruskal–Wallis test and one-way analysis of variance (ANOVA) revealed that plant identity had a significant effect on the OTU richness of endophytic fungi (*χ*^2^ = 17.849, *P* = 0.003), but not on that of epiphytic fungi (*F*_5,42_ = 0.654, *P* = 0.660). For example, the OTU richness of endophytic fungi was significantly higher in *E*. *agallocha* than in *B*. *gymnorrhiza* and *K*. *candel*, and higher in *A*. *marina* than in *K*. *candel* (Fig. 2).

### Community composition of epiphytic and endophytic fungi

Of the 635 fungal OTUs, 119 (18.7% of the total OTUs) were specific epiphytic fungi, 259 (40.8%) were specific endophytic fungi, and 257 (40.5%) shared between them (Additional file 2: Figure S3). For example, members of genera *Curreya*, *Peniophora*, and *Cytospora* were exclusively epiphytic, whereas members of genera *Auriculibuller*, *Yamadazyma*, *Pseudoplectania*, and *Simplicillium* were exclusively endophytic (Additional file 1: Table S1). Besides, the relative abundances of some abundant OTUs were significantly different between epiphytic and endophytic fungi in plant species (Additional file 3: Table S2).

**Fig. 3:**
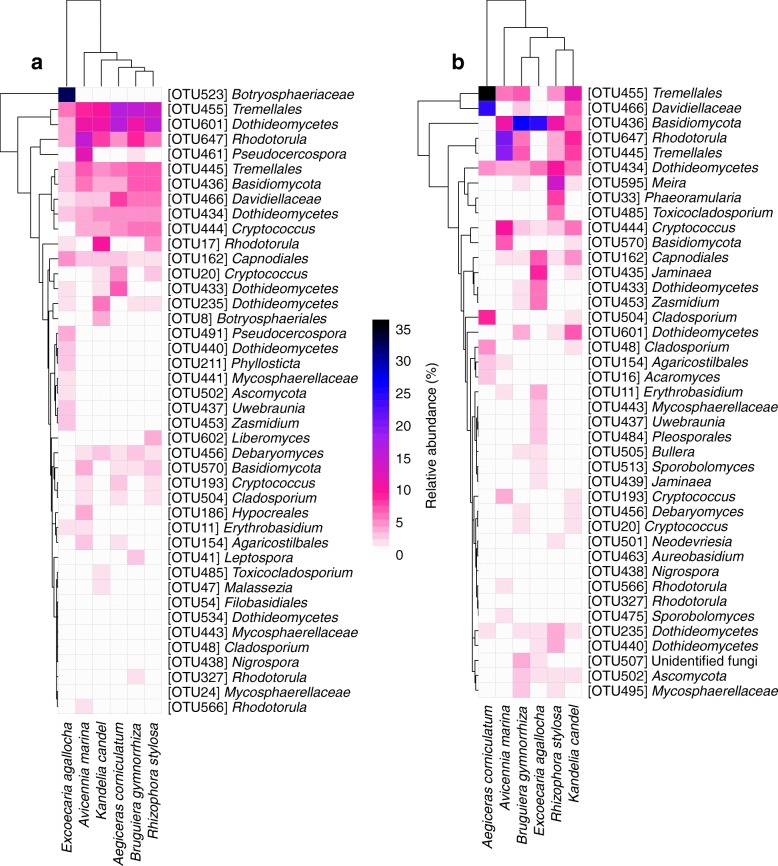
Heatmap depicting the distribution of relatively abundant fungal operational taxonomic units (OTUs, > 1000 reads) in mangrove species. **a** Epiphytic fungi. **b** Endophytic fungi. The color of each heat map cell indicates the relative abundance of the corresponding fungal OTUs. Cluster analysis was performed based on Bray–Curtis similarities

The heatmap revealed that the occurrence of some relatively abundant epiphytic and endophytic fungal OTUs was biased among plant species (Fig. 3a, b). For example, of the epiphytic fungi, three OTUs (*Hypocreales* OTU186, *Pseudocercospora* OTU461, and *Rhodotorula* OTU566) were distributed mainly in *A*. *marina*, while eight OTUs (*Phyllosticta* OTU211, *Uwebraunia* OTU437, *Dothideomycetes* OTU440, *Mycosphaerellaceae* OTU441, *Zasmidium* OTU453, *Pseudocercospora* OTU491, *Ascomycota* OTU502, and *Botryosphaeriaceae* OTU523) occurred mainly in *E*. *agallocha* (Fig. 3a). Among the endophytic fungi, five OTUs (*Acaromyces* OTU16, *Cladosporium* OTU48 and OTU504, *Tremellales* OTU455, and *Davidiellaceae* OTU466) occurred mainly in *A*. *corniculatum*, nine OTUs (*Erythrobasidium* OTU11, *Dothideomycetes* OTU433, *Jaminaea* OTU435 and OTU439, *Uwebraunia* OTU437, *Mycosphaerellaceae* OTU443, *Zasmidium* OTU453, *Pleosporales* OTU484, and *Sporobolomyces* OTU513) were present mainly in *E*. *agallocha*, and five OTUs (*Phaeoramularia* OTU33, *Dothideomycetes* OTU440, *Toxicocladosporium* OTU485, *Neodevriesia* OTU501, and *Meira* OTU595) occurred mainly in *R*. *stylosa* (Fig. 3b). In addition, indicator species analysis showed that there were 5 epiphytic fungal indicator OTUs, OTU461 (*Pseudocercospora*) for *A*. *marina* and 4 (*Phyllosticta* OTU211, *Botryosphaeriaceae* OTU216 and OTU523, and *Pseudocercospora* OTU491) for *E*. *agallocha*; by contrast, there were 30 endophytic fungal indicator OTUs, with 10 for *A*. *corniculatum*, 4 for *A*. *marina*, 11 for *E*. *agallocha*, and 5 for *R*. *stylosa* (OTUs taxonomic positions see Table 1).

**Table 1:** Epiphytic and endophytic fungal indicator operational taxonomic units (OTUs) in mangrove species

OTU	Taxonomic position	Mangrove species	*Indval* value (> 0.6)	*P* value
Epiphyte				
OTU461	*Pseudocercospora*	*Avicennia marina*	0.612	0.015
OTU211	*Phyllosticta*	*Excoecaria agallocha*	0.625	0.001
OTU216	*Botryosphaeriaceae*	*E*. *agallocha*	0.722	0.002
OTU491	*Pseudocercospora*	*E*. *agallocha*	0.687	0.001
OTU523	*Botryosphaeriaceae*	*E*. *agallocha*	0.863	0.001
Endophyte				
OTU29	*Neodevriesia*	*Aegiceras corniculatum*	0.625	0.001
OTU48	*Cladosporium*	*A*. *corniculatum*	0.765	0.001
OTU49	*Pestalotiopsis*	*A*. *corniculatum*	0.703	0.001
OTU109	*Capnodiales*	*A*. *corniculatum*	0.971	0.001
OTU130	*Capnodiales*	*A*. *corniculatum*	0.820	0.001
OTU355	*Agaricostilbales*	*A*. *corniculatum*	0.624	0.001
OTU356	*Dothideomycetes*	*A*. *corniculatum*	0.944	0.001
OTU357	*Cladosporium*	*A*. *corniculatum*	0.780	0.001
OTU466	*Davidiellaceae*	*A*. *corniculatum*	0.692	0.001
OTU504	*Cladosporium*	*A*. *corniculatum*	0.814	0.001
OTU15	*Capnodiales*	*Avicennia marina*	0.721	0.001
OTU25	*Basidiomycota*	*A*. *marina*	0.760	0.001
OTU102	*Sporidiobolales*	*A*. *marina*	0.861	0.001
OTU570	*Basidiomycota*	*A*. *marina*	0.619	0.001
OTU11	*Erythrobasidium*	*Excoecaria agallocha*	0.685	0.001
OTU433	*Dothideomycetes*	*E*. *agallocha*	0.605	0.001
OTU435	*Jaminaea*	*E*. *agallocha*	0.822	0.001
OTU437	*Uwebraunia*	*E*. *agallocha*	0.856	0.001
OTU439	*Jaminaea*	*E*. *agallocha*	0.919	0.001
OTU443	*Mycosphaerellaceae*	*E*. *agallocha*	0.658	0.001
OTU453	*Zasmidium*	*E*. *agallocha*	0.675	0.001
OTU484	*Pleosporales*	*E*. *agallocha*	0.686	0.001
OTU494	*Mycosphaerellaceae*	*E*. *agallocha*	0.747	0.001
OTU499	*Erythrobasidiales*	*E*. *agallocha*	0.625	0.002
OTU516	*Symmetrospora*	*E*. *agallocha*	0.658	0.001
OTU33	*Phaeoramularia*	*Rhizophora stylosa*	0.842	0.001
OTU42	*Zymoseptoria*	*R*. *stylosa*	0.625	0.001
OTU485	*Toxicocladosporium*	*R*. *stylosa*	0.722	0.001
OTU538	*Dothideomycetes*	*R*. *stylosa*	0.609	0.001
OTU595	*Meira*	*R*. *stylosa*	0.820	0.001

**Fig. 4:**
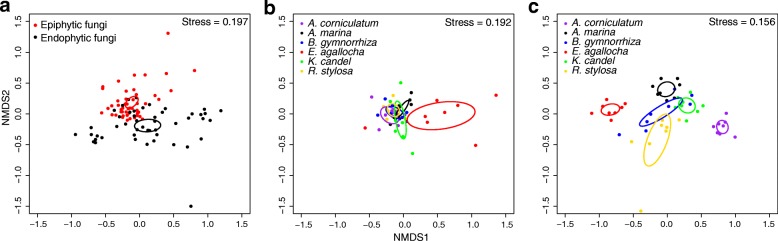
Non-metric multidimensional scaling (NMDS) ordination of the community composition of epiphytic and endophytic fungi in mangrove species. **a** Epiphytic and endophytic fungi. **b** Epiphytic fungi. **c** Endophytic fungi. Ellipses in the plots denote 95% confidence intervals for the centroids of epiphytic and endophytic fungi (**a**) and different mangrove species (**b**, **c**). *A*. *corniculatum*, *Aegiceras corniculatum*; *A*. *marina*, *Avicennia marina*; *B*. *gymnorrhiza*, *Bruguiera gymnorrhiza*; *E*. *agallocha*, *Excoecaria agallocha*; *K*. *candel*, *Kandelia candel*; *R*. *stylosa*, *Rhizophora stylosa*

The non-metric multidimensional scaling (NMDS) ordination revealed that the community composition of epiphytic and endophytic fungi was significantly different (Fig. 4a). Furthermore, the epiphytic fungal community composition in *E*. *agallocha* was significantly different from that in the other five plant species, but no significant difference among these latter five plant species was observed (Fig. 4b). The endophytic fungal community composition was significantly different in the six plant species, except for between *B*. *gymnorrhiza* and *K*. *candel* and between *B*. *gymnorrhiza* and *R*. *stylosa* (Fig. 4c). The permutational multivariate analysis of variance analysis (PerMANOVA) also indicated that the community composition of epiphytic and endophytic fungi was significantly different (*F* = 6.435, *R*^2^ = 0.064, *P* = 0.001). Furthermore, plant identity significantly affected the community composition of both endophytic fungi and epiphytic fungi, with a greater effect on endophytic fungi than on epiphytic fungi, as the variation explained by plant identity was higher for the endophytic fungal community (*F* = 8.125, *R*^2^ = 0.492, *P* = 0.001) than for the epiphytic fungal community (*F* = 2.648, *R*^2^ = 0.240, *P* = 0.001).

### Preference of plants and epiphytic and endophytic fungi

**Fig. 5:**
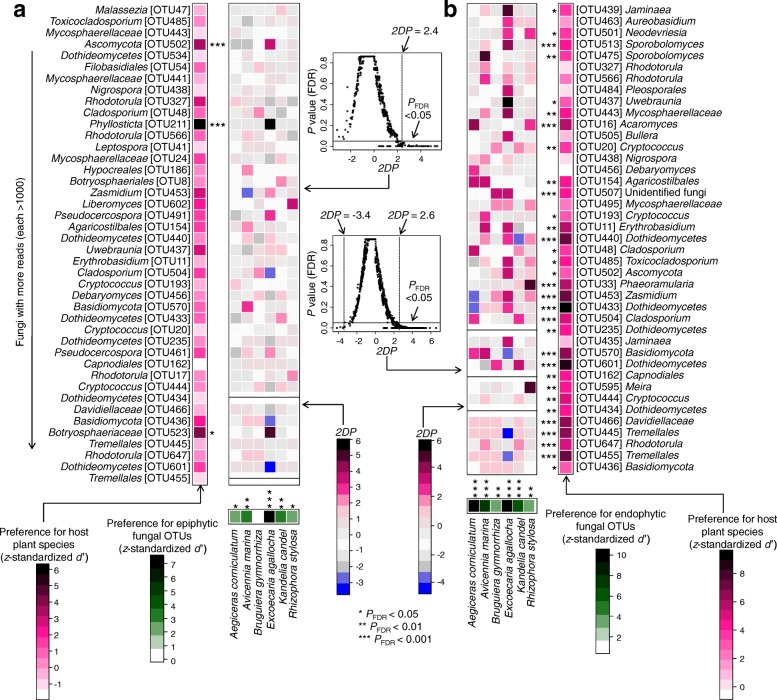
Preferences observed in plant-fungus associations. **a** Plant-epiphytic fungus association. **b** Plant-endophytic fungus association. The standardized *d*′ estimate of preferences for fungal operational taxonomic units (OTUs) is shown for each plant species (column). Likewise, the standardized *d*′ estimate of preferences for plant species is indicated for each of the observed fungal OTUs (row). Each cell in the matrix indicates a two-dimensional preference (*2DP*) estimate, which measures to what extent the association of a focal plant-fungus pair was observed more or less frequently than would be expected by chance. The *P* values were adjusted based on the false discovery rate (FDR). The relationship between *2DP* and FDR-adjusted *P* values shows that *2DP* values larger than 2.4 (epiphyte) and 2.6 (endophyte) and those smaller than − 3.4 (endophyte) represented strong preference and avoidance respectively (*P*_FDR_ < 0.05). Black line indicates that the 2DP value is not able to be calculated as the standard deviation of the number of samples for the focal plant-fungal OTU pair across randomized matrices is zero

Host/fungus preference analysis showed that five plant species (*A*. *corniculatum*, *A*. *marina*, *E*. *agallocha*, *K*. *candel*, and *R*. *stylosa*) had significant preferences for epiphytic fungi (Fig. 5a), but all six plant species showed significant preferences for endophytic fungi (Fig. 5b). In addition, 3 (*Phyllosticta* OTU211, *Ascomycota* OTU502, and *Botryosphaeriaceae* OTU523) out of the 42 relatively abundant epiphytic fungal OTUs showed significant host preferences (Fig. 5a). By contrast, 32 out of 41 relatively abundant endophytic fungal OTUs displayed significant host preferences, such as OTU16 (*Acaromyces*), OTU433, OTU440 and OTU601 (*Dothideomycetes*), OTU445 and OTU455 (*Tremellales*), OTU453 (*Zasmidium*), OTU466 (*Davidiellaceae*), and OTU570 (*Basidiomycota*) (Fig. 5b). In our dataset, 8 out of 240 pairs of plant species and epiphytic fungi showed significantly strong preferences (two-dimensional preferences (*2DP*) > 2.4); they included the pairs *E*. *agallocha* and OTU211 (*Phyllosticta*) and *A*. *marina* and OTU570 (*Basidiomycota*) (Fig. 5a). By contrast, 23 out of 228 pairs of plant species and endophytic fungi were observed to have significantly strong preferences (*2DP* > 2.6), such as *A*. *corniculatum* and OTU16 (*Acaromyces*), *E*. *agallocha* and OTU437 (*Uwebraunia*), and *R*. *stylosa* and OTU595 (*Meira*) pairs (Fig. 5b).

### Network structure of plant-epiphytic and plant-endophytic fungi

**Fig. 6:**
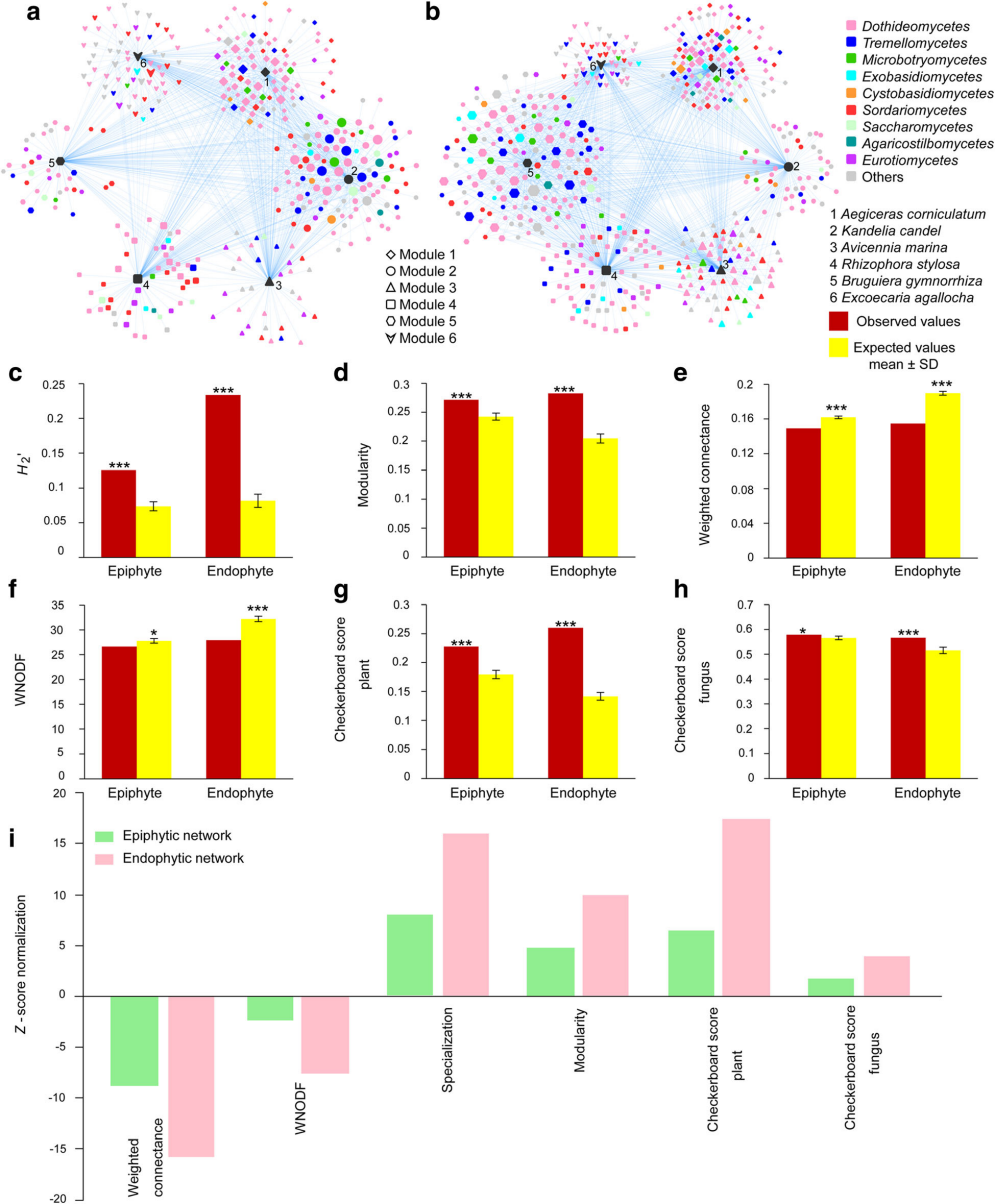
Architecture of the plant-fungus network. **a** Visualization of the epiphytic network. **b** Visualization of the endophytic network. In **a**, **b**, the size of nodes roughly represents the relative abundance of fungal operational taxonomic units. **c** *H*_2_′ metric of the network-level interaction specialization. **d** Barber's metric of modularity. **e** Weighted connectance. **f** Weighted nestedness metric based on overlap and decreasing fill (WNODF). **g** Checkerboard scores representing the extent to which the overlap of fungi is avoided in the plant community. **h** Checkerboard scores representing the extent to which the overlap of plants is avoided in the fungal community. **i** Standardized network properties with *Z*-score normalization of epiphytic and endophytic networks. Asterisks indicates significant differences between the observed and expected values according to *t* test (*P < 0.05; ***P < 0.001)

The network of plants and epiphytic and endophytic fungi is shown in Fig. 6a, b. The network structure of both plant-epiphytic and plant-endophytic fungi was highly specialized and modular but showed lowly connected and anti-nested properties. In particular, the observed values of *H*_2_′ and modularity were significantly higher than expected based on null models (Fig. 6c, d), whereas the observed values of weighted connectance and weighted nestedness metric based on overlap and decreasing fill (WNODF) were significantly lower than expected based on null models (Fig. 6e, f). The checkerboard scores analysis showed that the observed values for plant and fungal communities were significantly higher than those expected based on null models in both epiphytic and endophytic networks (Fig. 6g, h), indicating the existence of competitive interaction within the plant and fungal communities. Furthermore, the *Z*-score normalization analysis showed that the endophytic network was more highly specialized and more modular but less connected and more strongly anti-nested than the epiphytic network, and the degree of competitiveness within plant and fungal communities were greater in the endophytic network than in the epiphytic network (Fig. 6i).

## Discussion

This study showed that *Ascomycota* and *Basidiomycota* were relatively abundant in both epiphytic and endophytic fungal communities, which is in accordance with the findings of some previous studies using culture-independent methods [21, 47, 48]. However, previous studies using culture-based methods revealed that *Ascomycota* but not *Basidiomycota* were dominant in epiphytic and endophytic fungal communities [3, 23, 49, 50]. This difference may be ascribed to study methodology, as there are multiple challenges that limit the detection of *Basidiomycota* using traditional culture-based approaches [51]. For example, some *Basidiomycota* do not grow, or grow slowly, in culture in comparison to most *Ascomycota* and they are easily out-competed during the culturing process [3, 52, 53]. By contrast, culture-independent methods, such as high-throughput DNA sequencing techniques, can give a more complete picture of fungal communities compared with culture-based methods [47, 48, 51, 54]. We also found that *Dothideomycetes* and *Tremellomycetes* were dominant in epiphytic and endophytic fungal communities. In other studies, members of these fungal classes were also reported to be abundant in epiphytic [20, 48] and endophytic [47, 54, 55] fungal communities.

We found that plant identity had a significant effect on the OTU richness of endophytic fungi, but not on that of epiphytic fungi. Similarly, some previous studies have revealed that plant identity significantly influences the richness of endophytic fungi [14, 18], but not that of epiphytic fungi [20]. This difference may be because although different plant species receive the same fungal propagules on the leaf surface, endophytic fungi are filtered more by host plants than are epiphytic fungi [13]. For example, endophytic fungi need to penetrate host surfaces and absorb nutrients from hosts, and different plant species have various foliar physical characters and nutrient contents which can affect endophytic fungal colonization [17, 56]. By contrast, epiphytic fungi depend on nutrients deposited on leaves from the atmosphere or those exuded from leaves and are likely to be more affected by external environmental factors, such as wind speed, temperature, relative humidity, and solar radiation, rather than by the hosts [22].

This study showed that the community composition of epiphytic and endophytic fungi was significantly different, as reported in previous studies [13, 20–23, 50]. This difference may be caused by the distinct leaf microenvironments where epiphytic and endophytic fungi live, as mentioned above [13, 17, 22, 56]. In fact, we found that 18.7% and 40.8% of the total OTUs were unique epiphytic and endophytic fungi, respectively (Additional file 2: Figure S3). For example, members of genera *Curreya*, *Peniophora*, and *Cytospora* were exclusively epiphytic, whereas members of genera *Auriculibuller*, *Yamadazyma*, *Pseudoplectania*, and *Simplicillium* were restricted to the role of endophytic fungi (Additional file 1: Table S1). Similarly, members of *Yamadazyma*, *Pseudoplectania*, and *Simplicillium* were also reported as endophytic fungi in previous studies [46, 57, 58]. In addition, some relatively abundant fungal OTUs had biased occurrence in epiphytic and endophytic fungal communities, for example, some OTUs (e.g., *Rhodotorula* OTU17, *Phyllosticta* OTU211, and *Dothideomycetes* OTU601) were significantly relatively more abundant in epiphytic fungi than in endophytic fungi, while some OTUs (e.g., *Jaminaea* OTU439, *Neodevriesia* OTU501, and *Ascomycota* OTU502) were significantly relatively more abundant in endophytic fungi than in epiphytic fungi in this study.

We found that plant identity significantly affected the community composition of both epiphytic and endophytic fungi, as reported in some previous studies [14–18, 21]. Furthermore, plant identity had a greater effect on the community composition of endophytic fungi than that of epiphytic fungi in this study. One possible explanation may be that endophytic fungi are filtered more by host plants, whereas epiphytic fungi are more affected by environmental factors as mentioned above [13, 17, 22, 56]. In addition, our findings may be due to differences in host/fungus preferences, as our host/fungus preference analysis demonstrated that more endophytic fungal OTUs showed significant preferences for hosts than did epiphytic fungal OTUs (32 vs. 3), and more host species (6 vs. 5) showed significant preferences for endophytic fungi than for epiphytic fungi. In addition, more pairs of plant species and endophytic fungi than that of plant species and epiphytic fungi (23 vs. 8) showed significantly strong preferences. Moreover, our checkerboard scores analysis indicated that negative species interaction was stronger in endophytic than in epiphytic fungal communities, as competitive interaction can affect the fungal community assembly process, in that the presence of early-arriving fungal species has a negative influence on the ability of later-arriving species to colonize hosts [59].

Network analysis showed that both epiphytic and endophytic networks were characterized by high specialization and modularity, but low connectance and anti-nestedness. This pattern is similar with plant-endophytic fungus [41], orchid-mycorrhizal fungus [35, 36], ericaceous plant-fungus [40], and some plant-ectomycorrhizal fungus [32, 39] networks. Furthermore, the observed values of modularity and/or specialization were lower but that of nestedness was higher in epiphytic and endophytic networks than in ericaceous plant-fungus, orchid-mycorrhizal fungus, and some plant-ectomycorrhizal fungus networks [31, 32, 35, 36, 39, 40]. However, this pattern of epiphytic and endophytic networks differs from those of plant-arbuscular mycorrhizal fungus networks, which often tend to be highly nested and lowly specialized [33, 34, 37]. In addition, the observed values of connectance and nestedness were generally lower in epiphytic and endophytic networks than in plant-arbuscular mycorrhizal fungus networks [34, 39]. This difference may be ascribed to the varying degrees of connectance and specialization in different types of networks, as generally in biotrophic plant-fungal associations, nestedness increases but modularity decreases with increasing connectance [31], whereas nestedness decreases but modularity increases with increasing specificity [25].

Furthermore, we found that the endophytic network was more highly specialized and modular but less connected and more strongly anti-nested than the epiphytic network. The more strongly anti-nested pattern observed in the endophytic network compared to the epiphytic network indicates that partner-specific plant species are less likely to favor generalists over specialists among endophytic fungi than among epiphytic fungi [31]. Similarly, the higher degree of specialization and modularity in the endophytic network than in the epiphytic network suggests that plant hosts may select more strongly for a specific subset of endophytic partners than for a subset of epiphytic partners [42]. In addition, because modular organization can buffer communities against secondary extinctions following disturbance and increase overall network stability [30], the higher modularity of the endophytic network than that of the epiphytic network may suggest that the endophytic fungal community is more resistant to secondary species extinctions brought about by disturbance than is the epiphytic fungal community. In addition, we should realize that the sampling numbers in the present study are limited, which may not be enough for strong statistical analysis [60]. Therefore, more samples should be used in future study.

## Conclusion

This study provides the first example of research revealing differences in community and network structures of phyllosphere epiphytic and endophytic fungi associated with mangrove species using high-throughput DNA sequencing techniques. Plant identity had greater effects on the diversity and community composition of endophytic fungi than that of epiphytic fungi. Network analysis showed the endophytic network was more highly specialized and modular but less connected and more strongly anti-nested than epiphytic network. Our findings support a more intimate relationship between plants and endophytic fungi than between plants and epiphytic fungi, and suggest that the endophytic fungal community is more resistant to environmental disturbance than the epiphytic fungal community in the mangrove forest ecosystem. This study may help us to deeper understand mechanisms underlying species coexistence and community stability in ecosystems.

## Methods

### Study site and sampling

The study was conducted in the Zhanjiang Mangrove National Nature Reserve in south China (21° 32′ N, 109° 46′ E, 20,278.8 ha, 2–7 m above sea level). This study site is located in a tropical climate zone, with an annual mean temperature of 23.4 °C, and an annual mean precipitation of 1600 mm, occurring mostly from April to September. There are six mangrove species, *Aegiceras corniculatum* (*Myrsinaceae*), *Avicennia marina* (*Verbenaceae*), *Bruguiera gymnorrhiza*, *Kandelia candel* and *Rhizophora stylosa* (*Rhizophoraceae*), and *Excoecaria agallocha* (*Euphorbiaceae*), at the site. On 15 March 2015, we selected eight individuals (replicates) of each of the six plant species, each > 50 m away from all other members of the same plant species. Subsequently, 30 healthy leaves were randomly collected from each plant individual, and immediately placed in sterile plastic bags, labeled, and transported to laboratory in an ice-box. In total, 48 leaf samples were used in this study. All the samples were stored at − 80 °C until required for DNA extraction.

### Molecular analysis

Epiphytic fungi were washed from leaf surfaces according to Gourion et al. [61]. Frozen leaves (5.0 g) were transferred into a 50-mL plastic tube filled with sterile cooled TE buffer (10 mM Tris–HCl, 1 mM EDTA, pH 7.5) and then subjected to alternating sonication (45 s) and vortexing (30 s) three times. The leaves were transferred to new tubes and the suspension was centrifuged at 10,000×*g* for 10 min. The supernatant was discarded, then the pellet was resuspended in 5 mL cetyltrimethylammonium bromide (CTAB) extraction buffer (2% (*w*/*v*) CTAB, 100 mM Tris–HCl, 1.4 M NaCl, 20 mM EDTA, 1.5% PVP, 0.5% 2-mercaptoethanol, pH 8.0) preheated to 65 °C, and homogenized at 6.0 m/s for 30 s in a FastPrep®-24 Instrument (MP Biomedicals, Illkirch, France) according to the manufacturer’s instructions. For endophytic fungi, the leaf samples described above were surface sterilized by consecutive immersion for 1 min in 75% ethanol, 3 min in 3.25% sodium hypochlorite, and 30 s in 75% ethanol, followed by three rinses in sterile distilled water [49]. Subsequently, the treated leaves were freeze-dried using liquid nitrogen and homogenized using a sterilized mortar and pestle and then transferred to a tube with 5 mL CTAB extraction buffer preheated to 65 °C. Genomic DNA of epiphytic and endophytic fungi was extracted using the modified CTAB method [49]. The DNA concentration was measured using a NanoDrop 1000 Spectrophotometer (Thermo Scientific, Wilmington, USA).

The fungal internal transcribed spacer 2 (ITS2) region of rRNA gene was amplified for high-throughput Illumina MiSeq sequencing using a two-step PCR procedure. The initial amplification of the entire ITS region with primers ITS1F [62] and ITS4 [63] was carried out in a 25 μL reaction solution containing 2.5 μL 10 × buffer, 1.5 mM MgSO_4_, 250 μM of each dNTP, 0.75 μM of each primer, 0.5 U KOD-plus-Neo polymerase (Toyobo, Tokyo, Japan), and 10 ng of template DNA. The PCR conditions were set at 94 °C for 5 min, 30 cycles for denaturation at 94 °C for 1 min, annealing at 54 °C for 50 s, and extension at 68 °C for 1 min, followed by a final extension at 68 °C for 10 min. The product of the first PCR was diluted by a factor of 50 with sterile deionized water and 1.0 μL of the diluted solution was used as a template for the second PCR with the same conditions as the first amplification, except that the primers used were fITS7 [64] and ITS4 linked with 12-base barcode sequences. Furthermore, the sterile water used in the final rinse was subjected to the two-step PCR as negative controls to test the efficacy of surface sterilization. No PCR products were detected demonstrating the effectiveness of the surface sterilization procedure. We also used sterile deionized distilled water as negative controls in all steps of the PCR procedure to test the presence of contamination in reagents. No bands were observed in all negative controls. The PCR products were purified using a PCR Product Gel Purification Kit (Bioteke, Beijing, China), and 50 ng purified DNA of each of the 96 samples was pooled and adjusted to 10 ng μL^−1^. A sequencing library was generated by addition of an Illumina sequencing adaptor (5′-GATCGGAAGAGCACACGTCTGAACTCCAGTCACATCACGATCTCGTATGCCGTCTTCTGCTTG-3′) to the product using an Illumina TruSeq DNA PCR-Free Library Preparation Kit (Illumina, CA, USA), following the manufacturer’s instructions. The library was applied to an Illumina MiSeq PE 250 platform for sequencing using the paired end option (2 × 250 base pairs (bp)) at the Environmental Genome Platform of Chengdu Institute of Biology, Chinese Academy of Sciences, China.

### Bioinformatics analysis

Raw sequences were filtered using Quantitative Insights into Microbial Ecology (QIIME) version 1.7.0 [65] to eliminated low-quality sequences, defined as those with an average quality score < 20, without valid primer sequence or barcode sequence, containing ambiguous bases, or length < 250 bp. Of the remaining sequences, the ITS2 region was extracted using the fungal ITSx software package [66], and potential chimeras were subsequently checked using the chimera.uchime command in MOTHUR version 1.31.2 [67] by comparison to entries in the unified system for the DNA based fungal species linked to the classification (UNITE) database [68]. The non-chimeric sequences were clustered into operational taxonomic units (OTUs) at a 97% sequence similarity level based on the UPARSE pipeline using USEARCH version 8.0 [69] after dereplication and discarding all singletons. A representative sequence (the most abundant) of each OTU was selected for searching against the UNITE database version 18.11.2018 [70] using the sintax function [71] in USEARCH with a confidence cut-off (*P*) value of 0.65. We then excluded the OTUs with < 10 reads from all the samples as their sequences could contain PCR or sequencing errors [72]. To eliminate the effects of different sequence numbers among the samples on the fungal community identified, the number of sequences per sample was normalized to the smallest sample size using the sub.sample command in MOTHUR. The representative fungal OTU sequences have been submitted to the European Nucleotide Archive (ENA) under study accession number PRJEB24460. Detailed information about fungi in the present study is given in Additional file 1: Table S1.

### Data analysis

All the statistical analyses were implemented in R version 3.3.2 [73], except for the network visualization, which was carried out in CYTOSCAPE version 3.4.0 [74], and modularity analysis, which used the program MODULAR [75]. Fungal OTU richness is defined as the number of fungal OTUs in a sample. The relative abundance of a specific fungal OTU and class is defined as the number of reads of that OTU and class as a percentage of the number of all reads in a sample. One-way analysis of variance (ANOVA) was used to explore the effect of plant identity on fungal OTU richness after the square root or log transformation as the data did not satisfy the normality of distribution and homogeneity of variance, then significant differences between plant species were further compared using Tukey’s honestly significant difference (HSD) test at *P* < 0.05. As the data of endophytic fungal OTU richness did not satisfy the normality of distribution and homogeneity of variance after the square root and log transformation, then non-parametric Kruskal–Wallis test was used, followed by Conover’s test for multiple comparisons using the posthoc.kruskal.conover.test function in the PMCMR package [76]. The relative abundances of abundant epiphytic and endophytic fungal OTUs (> 1000 reads) among different plant species were depicted using the pheatmap function in the pheatmap package version 1.0.8 [77]. Furthermore, the relative abundance of abundant OTUs between the epiphytic and endophytic fungi in each plant species was compared using paired *t* test if the data satisfy the normality of distribution after the root square or log transformation; otherwise, paired Wilcoxon signed rank test was used. In addition, we conducted indicator species analysis of fungal OTUs for each plant species based on the OTU relative abundance (*indval* value > 0.6 and *P* < 0.05 are strong indicators for a species) using the indval function in the labdsv package version 1.8-0 [78].

The distance matrices of community composition (Hellinger-transformation of the OTU read data) of epiphytic and endophytic fungi were constructed by calculating dissimilarities using the Bray–Curtis method [79]. Subsequently, non-metric multidimensional scaling (NMDS) was used to visualize the community composition dissimilarities of epiphytic and endophytic fungi using the metaMDS command in the vegan package version 2.4-1 [80]. In order to evaluate the effect of plant identity on the community composition of epiphytic and endophytic fungi, permutational multivariate analysis of variance (PerMANOVA) was carried out, using the adonis command in the vegan package, based on 999 permutations [80]. The rarefaction curves of the OTUs of epiphytic and endophytic fungi observed in each plant species were calculated using the specaccum function in the vegan package [80].

Host/fungus preference analysis was carried out according to Toju et al. [40]. In brief, the sample (row) × fungal OTU (column) data matrix (in which a cell entry depicts the number of reads of an OTU in a sample) was binarized to a sample-level matrix (present-absent data) and then converted into a species-level matrix (quantitative data), in which rows denote plant species, columns represent fungal OTUs, and cell entries indicate the number of samples from which respective combinations of plants and fungi were observed. By shuffling the plant species labels in the sample × fungal OTU matrix, a randomized species-level matrix was obtained based on 1000 permutations. The interaction specialization index (*d*′) [81] was calculated using the dfun function in the bipartite 2.05 package [82]. The *d*′ value of each fungal OTU was standardized as follows: standardized *d*′ = [*d*′_observed_ − Mean (*d*′_randomized_)]/SD (*d*′_randomized_), where *d*′_observed_ is the *d*′ estimate of the original data, and Mean (*d*′_randomized_) and SD (*d*′_randomized_) are the mean and standard deviation of the *d*′ values of the randomized data matrices. The standardized *d*′ of each plant species was also calculated based on the original and randomized data matrices as mentioned above. Because it is difficult to estimate the host preferences of rare fungi, the *d*′ estimates of the relatively abundant epiphytic and endophytic fungal OTUs (> 1000 reads) in the sample × fungal OTU matrix are shown. In addition, based on the species-level original and randomized matrices, we quantified two-dimensional preferences (*2DP*) to evaluate to what extent each pair of a plant species (*i*) and fungal OTU (*j*) was observed (counts) more or less frequently than would be expected by chance. *2DP* (*i*, *j*) = [*N*_observed_ (*i*, *j*) − Mean (*N*_randomized_ (*i*, *j*))]/SD (*N*_randomized_ (*i*, *j*)), where *N*_observed_ (*i*, *j*) is the number of the samples from which a pair of a plant species and a fungal OTU was observed in the original data, and Mean (*N*_randomized_ (*i*, *j*)) and SD (*N*_randomized_ (*i*, *j*)) are the mean and the standard deviation of the number of samples for the focal plant-fungal OTU pair across randomized matrices. The *P* value obtained from the preference analysis was adjusted based on the false discovery rate (FDR) [83].

To visualize network structure for the epiphytic and endophytic fungi, we drew a network based on the species-level matrices using the prefuse force directed opencl layout in CYTOSCAPE version 3.4.0 [74]. We then examined the architectural properties of the epiphytic and endophytic networks based on the species-level matrices according to Toju et al. [40]. To perform a randomization test, randomized matrices were obtained based on the shuffle-sample null model with 1000 permutations. The network indices used in the analysis were the weighted connectance [84], the *H*_2_′ metric of network-level specialization [81], Barber’s metric of bipartite network modularity [85], the weighted nestedness metric based on overlap and decreasing fill (WNODF) [86], and checkerboard scores [87] representing the degree to which overlap of partners was avoided within the plant/fungus community. Calculations of the weighted connectance, *H*_2_′, WNODF, and checkerboard scores were performed based on the species-level original and randomized matrices using the networklevel command in the bipartite package [82]. For modularity analysis, the species-level original and randomized matrices were binarized and output from R. Subsequently, the binary data were analyzed in the MODULAR program for simulated annealing-based estimation of network modularity [88]. Next, *t* tests were used to examine the difference between the observed and the random values at *P* < 0.05. To make comparisons across networks, the network indices were standardized with *Z*-score normalization which can correct for variation in species richness and the number of interactions [89]. The *Z*-score is defined as *Z* = (*E*_observed_ − *E*_randomized_)/SD_randomized_, where *E*_observed_ is the observed value and *E*_randomized_ and SD_randomized_ are the mean value and the standard deviation of the randomized matrices [89].

## Additional files

Additional file 1: **Table S1.** Molecular identification of epiphytic and endophytic fungi at a 97% sequence identity level in this study. (XLSX 113 kb)

Additional file 2: **Figure S1.** Ranking by abundance of the observed fungal operational taxonomic units (OTUs). a Epiphytic fungi. b Endophytic fungi. **Figure S2.** Rarefaction curves for the observed fungal operational taxonomic units (OTUs) in mangrove species. a Epiphytic fungi. b Endophytic fungi. *A*. *corniculatum*, *Aegiceras corniculatum*; *A*. *marina*, *Avicennia marina*; *B*. *gymnorrhiza*, *Bruguiera gymnorrhiza*; *E*. *agallocha*, *Excoecaria agallocha*; *K*. *candel*, *Kandelia candel*; *R*. *stylosa*, *Rhizophora stylosa*. **Figure S3.** Venn diagram showing the number of specific and shared operational taxonomic units (OTUs) of epiphytic and endophytic fungi. The percentage of these OTUs accounting for the total number of OTUs shows in parenthesis. (DOCX 127 kb)

Additional file 3: **Table S2.** Comparison of the relative abundance of abundant operational taxonomic units (OTUs, > 1000 reads) between epiphytic and endophytic fungi. (XLSX 16 kb)
