# Supplementary material for: Phyllosphere epiphytic and endophytic fungal community and network structures differ in a tropical mangrove ecosystem
Source: Microbiome. 2019 Apr 9;7:57. doi: 10.1186/s40168-019-0671-0 (PMC6456958; doi:10.1186/s40168-019-0671-0)
Supplement: Supplementary file 2 — Figure S1. Ranking by abundance of the observed fungal operational taxonomic units (OTUs). a Epiphytic fungi. b Endophytic fungi. Figure S2. Rarefaction curves for the observed fungal operational taxonomic units (OTUs) in mangrove species. a Epiphytic fungi. b Endophytic fungi. A. corniculatum, Aegiceras corniculatum; A. marina, Avicennia marina; B. gymnorrhiza, Bruguiera gymnorrhiza; E. agallocha, Excoecaria agallocha; K. candel, Kandelia candel; R. stylosa, Rhizophora stylosa. Figure S3. Venn diagram showing the number of specific and shared operational taxonomic units (OTUs) of epiphytic and endophytic fungi. The percentage of these OTUs accounting for the total number of OTUs shows in parenthesis. (DOCX 127 kb) [file 40168_2019_671_MOESM2_ESM.docx]

**Phyllosphere epiphytic and** **endophytic fungal community and network structures differ in a tropical mangrove ecosystem**

**Hui Yao****^1,2^, Xiang Sun^1^, Chao He^3^,** **Pulak Maitra^1,2^,** **Xing-chun Li^1^, and Liang-dong Guo^1,2*^**

^1^State Key Laboratory of Mycology, Institute of Microbiology, Chinese Academy of Sciences, Beijing 100101, People’s Republic of China

^2^College of Life Sciences, University of Chinese Academy of Sciences, Beijing 100049, People’s Republic of China

^3^Institute of Medicinal Plant, Chinese Academy of Medical Sciences and Peking Union Medical College, Beijing 100193, People’s Republic of China

Email addresses

Hui Yao: [yaohui1428@163.com](mailto:yaohui1428@163.com)

Xiang Sun: [sunx@im.ac.cn](mailto:sunx@im.ac.cn)

Chao He: [hc891215@126.com](mailto:hc891215@126.com)

Pulak Maitra: [3305808731@qq.com](mailto:3305808731@qq.com)

Xing-chun Li: [lixc@im.ac.cn](mailto:lixc@im.ac.cn)

***** Correspondence

Liang-dong Guo

Email: [guold@im.ac.cn](mailto:guold@im.ac.cn)


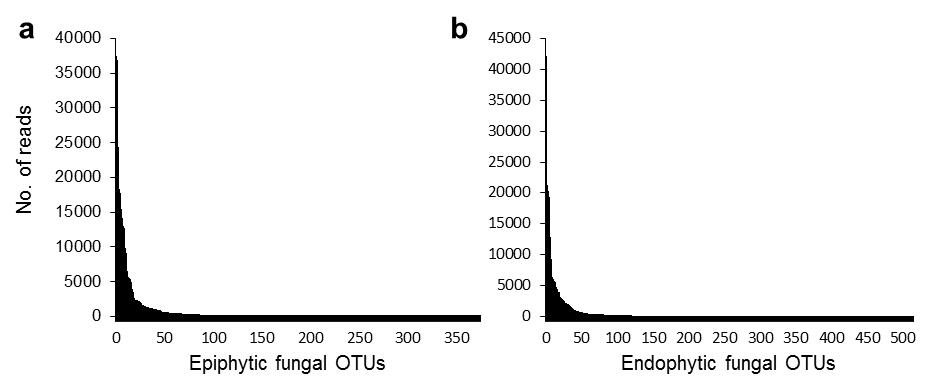


**Figure S1.** Ranking by abundance of the observed fungal operational taxonomic units (OTUs). **a** epiphytic fungi. **b** endophytic fungi.

**Figure S2.** Rarefaction curves for the observed fungal operational taxonomic units (OTUs) in mangrove species. **a** epiphytic fungi. **b** endophytic fungi. *A. corniculatum*, *Aegiceras corniculatum*; *A. marina*, *Avicennia marina*; *B. gymnorrhiza*, *Bruguiera gymnorrhiza*; *E. agallocha*, *Excoecaria agallocha*; *K. candel*, *Kandelia candel*; *R. stylosa*, *Rhizophora stylosa*.

**Figure S3.** Venn diagram showing the number of specific and shared operational taxonomic units (OTUs) of epiphytic and endophytic fungi. The percentage of these OTUs accounting for the total number of OTUs shows in parenthesis.
